# Cataract complications

**Published:** 2008-03

**Affiliations:** Consultant Ophthalmologist, Tennent Institute of Ophthalmology, Gartnavel Hospital, 1053 Great Western Road, Glasgow G12 0YN, Scotland.

**Figure:**
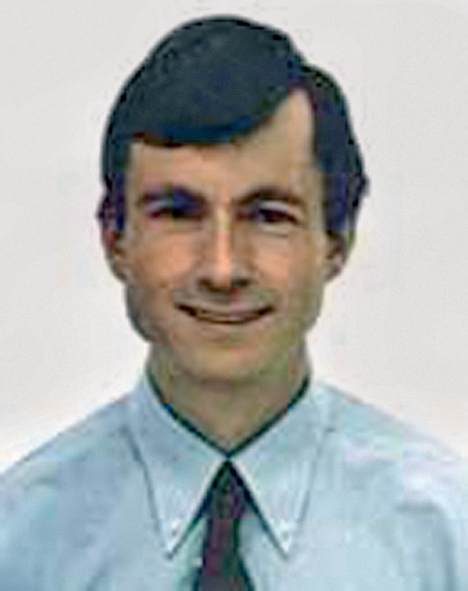


Any eye surgeon, no matter how experienced, will occasionally encounter a serious cataract complication. Although complications may be devastating for the patient and are always distressing for the surgeon, are they really a major issue for VISION 2020? The evidence says that they are.

## Impact

We know from numerous population-based surveys that a significant number of cataract operations may have poor outcomes (defined as presenting visual acuity of less than 6/60).

Poor outcomes are distressing or disappointing for patients. They reflect badly on the health or surgical facility and on the surgical team. Poor outcomes may also affect the sustainability of services; they discourage other patients from coming for surgery and make patients even more reluctant to contribute towards the cost of cataract operations.

In general, poor vision after cataract surgery is caused by: inadequate correction of post-operative refractive error (lack of **spectacles**); failure to detect pre-existing eye conditions, e.g. macular degeneration or amblyopia (**selection**); or surgical complications (**surgery**).

The widespread adoption of intraocular lenses is starting to decrease the number of patients left functionally blind after cataract surgery because they are not able to obtain the necessary aphakic correction spectacles.

**Figure:**
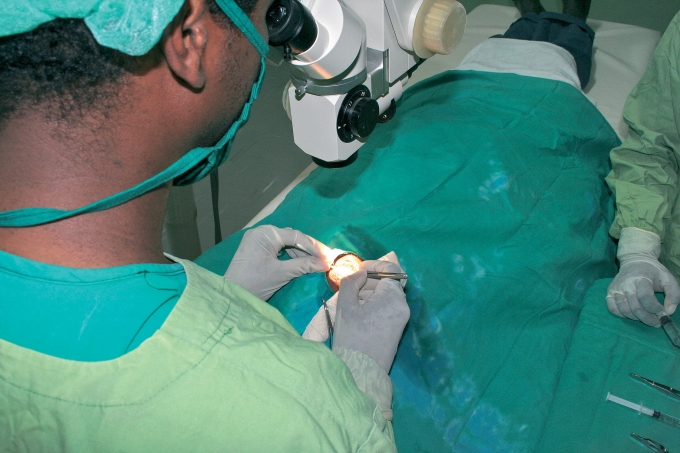
**Cataract surgery. ETHIOPIA**

Problems of selection can be addressed by careful pre-operative evaluation, which should reduce the number of poor results due to the presence of other eye diseases. This will help to prevent complications.

Surgical complications, which are the main focus of this issue, can to some extent be prevented by good practice and surgical technique. When complications do occur, proper management is crucial to reduce the possibility of a poor outcome for the patient.

There are currently no comprehensive figures on the proportion of poor outcomes of cataract surgery in developing countries and on the relative importance of spectacles, selection, and surgery (Table 1, page 2, provides data from Bangladesh,^1^ Kenya,^2^ and Pakistan^3^). At a conservative estimate, at least 25% (or 1.5 million) of the six million cataract operations performed annually in developing countries will have poor outcomes. About one quarter of these poor outcomes are due to surgical complications. Over 375,000 people can therefore suffer permanent visual impairment every year as a result of surgical complications.

**Table 1.** ***Causes of poor outcomes (presenting vision <6/60)***

	Percentage of total number of operations leading to a poor outcome	Cause of poor outcomes		
		Spectacles	Selection	Surgery
Bangladesh	28%	37%	41%	22%
Kenya	22%	34%	36%	30%
Pakistan	34%	36%	39%	25%

This means that surgical complications, and cataract complications in general, represent a significant obstacle to the success of any blindness prevention programme. The topics discussed in this issue are therefore vital to the successful implementation of VISION 2020.

## Important complications

Many things can go wrong during or immediately after cataract surgery. It is impossible to address every single complication in one issue of the journal, so we have concentrated on those that we feel are important.

What is an important complication? Some complications are common, but their impact is relatively minor. Others are rare but have a devastating impact. The articles in this issue will focus primarily on capsular rupture and vitreous loss, which is relatively common and potentially serious, and on endophthalmitis, which is rare but devastating.

Does capsular rupture and vitreous loss matter? Even in well-equipped teaching hospitals in the United Kingdom, vitreous loss is associated with a nearly fourfold greater risk of a poor visual outcome.^4^ In operating theatres without vitrectomy equipment, the risk of a poor outcome is likely to be even higher. However, not every patient who suffers capsular rupture and vitreous loss experiences a poor outcome. If the complication is managed well, it is possible to retain excellent vision (see article on page 6).

In high-income countries, the incidence of capsular rupture and vitreous loss appears to be declining and is now in the region of 1–2%. This improvement may be related to the use of phacoemulsification and to earlier intervention, which means that the great majority of cataracts are now removed before they are mature. In low- and middle-income countries, however, the incidence of capsular rupture and vitreous loss appears to be higher.^5^ This is probably due to the greater complexity of many cataract operations in developing countries, rather than to specific deficiencies of training, expertise, or equipment used.

Vitreous loss also increases the risk of endophthalmitis, the most feared complication of intraocular surgery. The incidence of endophthalmitis may vary. Studies from Europe give the estimated incidence as 0.14%.^6^ At Aravind Eye Hospital, in India, this incidence is about 0.05%.^7^

The causes of endophthalmitis might vary with geography. In most European studies, *Staphylococcus epidermidis* is the most common infecting microorganism. This bacterium is found in normal eyelid skin and conjunctiva, and it enters the eye during surgery. However, in South India, *Nocardia* species were the commonest cause of infection.^7^ When endophthalmitis does occur, the prognosis is grim. In the UK, one third of patients who suffered this complication had a final visual acuity (VA) of less than 6/60, and 13% had lost all light perception.^6^ At Aravind Eye Hospital in India, 65% of eyes had VA <6/60.^7^ However, these figures also show that the prognosis following endophthalmitis is by no means hopeless.

## Preventing complications

We know that certain eyes are more likely to suffer complications than others (see article on page 12). It is therefore very important to detect these conditions before surgery. For example, eyes with endothelial dystrophy (such as Fuch's dystrophy and corneal dystrophy), pseudoexfoliation, mature cataracts, or high ametropia (>6 dioptres of myopia or hypermetropia) are all at greater risk than eyes without these features. Simple scoring systems have been devised to stratify patients into low, medium, and high risk.^8^

It is important to collect data in order to identify patients at risk and to monitor their management before and after surgery. Even where the incidence of complications is low, regular collection of data helps to identify high-risk patients and to confirm that they are being managed appropriately. Monitoring of cataract surgical outcomes is associated with a reduction in the incidence of surgical complications.^9^

Some risk factors are intrinsic to the patient and, short of avoiding surgery altogether, very little can be done to eliminate them. However, in the event of surgery, high-risk cases should be operated on in an appropriate setting, by a surgeon who has the right level of experience. It has been shown that surgery carried out in eye camps, or by an inexperienced trainee, is more likely to result in complications than surgery undertaken in hospital by an experienced surgeon. Therefore, if patients with high-risk eyes are identified, they should be operated on by a fully trained surgeon, preferably in a base hospital.

Although intrinsic risk factors cannot be avoided, other factors which may increase the risk of surgical complications are related to the delivery of the surgery. These latter risks can, and should, be modified. Much can be done before and during surgery to reduce the rate of complications.

Meticulous sterilisation of all surgical instruments and fluids, and careful aseptic technique, are of course essential. Articles in this issue describe important steps to avoid complications during small incision cataract surgery (page 4) and how to reduce the risk of endophthalmitis (page 9). Recently, a large randomised clinical trial has shown a substantial reduction in the risk of endophthalmitis if 1 mg of cefuroxime is injected into the anterior chamber at the conclusion of surgery (see abstract and comment on page 11). This technique should be adopted universally, as it has the potential to save the sight of thousands of people per year.

## The importance of managing complications

With all complications, including capsular rupture and vitreous loss, and even endophthalmitis, the prognosis is better if the complication is managed effectively. Not every patient who suffers capsular rupture and vitreous loss experiences a poor outcome. If the complication is managed well, it is possible for the patient to retain excellent vision. However, we often do not deal with vitreous loss as well as we should. The article on page 6 provides top tips from experienced cataract surgeons for managing vitreous loss. In the case of endophthalmitis, early recognition and prompt treatment with intravitreal vancomycin and either ceftazidime or amikacin seems to offer the best hope of visual recovery. With immediate use of intravitreal antibiotics, some eyes will recover useful vision.

Because complications can and will occur, even in the best of cases, the eye care team must be prepared to manage them efficiently. Being prepared means: being trained to manage the problem; knowing where the relevant supplies are kept; having the right drugs and equipment on hand; and ensuring that the entire team is aware of the protocols for dealing with a complication. For example, there should be a protocol for vitrectomy in case of vitreous loss, and appropriate equipment should be on site. If phacoemulsification is being used, a protocol is needed to deal appropriately with dropped nuclei. When this complication is managed by prompt vitrectomy and fragmentation of the nucleus, the outcomes are normally good. However, if the nuclear material is not removed, the eye will be blinded by a combination of severe inflammation and glaucoma. No eye clinic should be using phacoemulsification unless they have identified a facility to which they can refer patients for vitrectomy and fragmentation of a retained nucleus. As phacoemulsification becomes more common in low- and middle-income countries, the number of dropped nuclei will also increase. Dislocation of fragments of the lens nucleus into the vitreous occurs in approximately 0.3% of phacoemulsification operations. The incidence may be higher in low- and middle-income countries, where dense cataracts and pseudoexfoliation are more common.^10^

The management of complications needs to be incorporated into training programmes. For example, management of vitreous loss, like every other surgical skill, can only be learnt by practicing under the supervision of a more experienced surgeon. However, although vitreous loss is most likely to occur while the surgeon is inexperienced, when it does occur, the trainer will usually take over. This means that, in some developed countries, ophthalmologists may do a few hundred cataract operations during their training, but will only manage vitreous loss two or three times.

**Figure:**
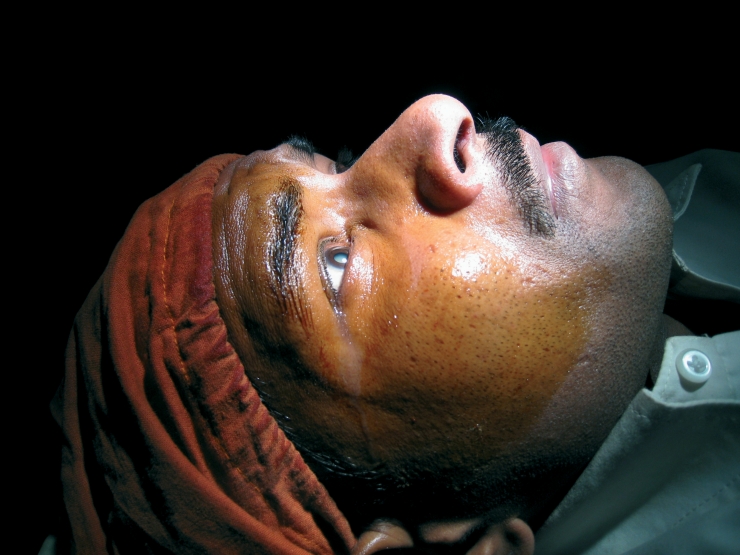
**Skin cleaned with povidone-iodine (Betadine 10%) before a cataract operation. NEPAL**

Our training programmes rightly emphasise the avoidance of complications in cataract surgery. However, we need a greater emphasis on the correct management of these complications when they do occur, as they inevitably will. No trainee is truly competent to operate on cataract patients independently unless, for example, they are also competent in the management of vitreous loss.

## Conclusion

In conclusion, the surgeon's first responsibility is to prevent complications. However, despite our best efforts, they will occur. Our next priority is to ensure that we are prepared to deal with these complications effectively so that our patients can obtain good vision, regardless of what went wrong during surgery. If we improve our management of complications, we can be certain that we will reduce the number of poor visual outcomes and disappointed cataract patients.

In striving to reach the goals of VISION 2020, we must be careful to maintain a culture that values outcome (the quality of cataract operations) as highly as output (the number of operations performed).
